# Promises and challenges of artificial intelligence in haematological diagnostics

**DOI:** 10.1111/bjh.70055

**Published:** 2025-07-28

**Authors:** Jan‐Niklas Eckardt, Jan Moritz Middeke

**Affiliations:** ^1^ Department of Internal Medicine I, University Hospital Carl Gustav Carus TUD Dresden University of Technology Dresden Germany; ^2^ Else Kröner Fresenius Center for Digital Health TUD Dresden University of Technology Dresden Germany

**Keywords:** AI literacy, artificial intelligence, diagnostics, explainable artificial intelligence, haematology

## Abstract

Artificial intelligence (AI) offers the promise of automating key tasks in haematology and unveiling novel biomarkers. However, model outputs may be biased, lack interpretability or fail to generalise. AI literacy is essential for haematologists to critically evaluate model outputs, effectively using them as decision support tools.

## Graphical abstract

Artificial intelligence (AI) promises to transform haematological diagnostics through automation, accuracy, scalability and novel biomarker discovery. However, dataset bias, lack of interpretability and challenges in model generalisation highlight the need for AI literacy and regulatory oversight to ensure safe and effective clinical integration.

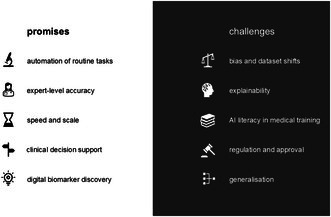

## WHERE ARTIFICIAL INTELLIGENCE MEETS HAEMATOLOGY

In 2012, Krizhevsky et al.^1^ demonstrated that deep learning is capable of highly accurate image classification, sparking a revolution in image processing. In healthcare, artificial intelligence (AI) systems provide fast and accurate diagnoses in a scalable manner, often performing on par with or outperforming human experts. Thus, they may help improve diagnostic accuracy and ease the burdens in overextended healthcare systems in light of demographic developments. Unlike conventional software, AI models learn from training data rather than relying on predefined rules. For example, the strength of the connections between artificial neurons in a neural network is determined during the training stage as numerical values (weights) in a similar process to neural plasticity in the human neocortex. AI has shown tremendous potential in automating tasks otherwise requiring human experts, such as cell or image classification in cytomorphology, interpreting results from multicolour flow cytometry, karyotyping, genetic variant interpretation and identification of genomic profiles (comprehensively reviewed by Walter et al.^2^). Clinical decision support systems (CDSS)—software tools aggregating information for healthcare providers to support or guide decision‐making processes—can stratify patients to data‐driven risk groups based on genomic signatures^3^ or predict treatment response.^4^, ^5^ However, despite their promise, these systems face several challenges every haematologist should be aware of to make safe and informed clinical decisions.

## BEWARE BIASES: AI IS ONLY AS GOOD AS ITS TRAINING DATA

As AI models rely on the quantity and quality of training data, their training and subsequently their outputs can be heavily skewed by biases within the training data. Models trained on narrow datasets risk poor transferability when encountering out‐of‐distribution samples. This is particularly relevant in high‐stake settings such as haematology, where AI tools not trained on rare disease subtypes may misclassify them. Furthermore, labelling data often require human experts, introducing subjectivity which potentially limits model robustness. Consequently, many AI models in healthcare currently struggle to generalise beyond their original data source.^6^ One may pretrain a model on a similar dataset and transfer it to a haematological task where it is fine‐tuned: For instance, image classifiers are commonly pretrained on image databases such as ImageNet^7^ to initialise weights for object detection, and subsequently transferred to, for example, microscopic images for fine‐tuning. Changes in the contextual environment of an AI model—different demographics, changes in the patient population or events such as a global pandemic—should incentivise the user to update their models to mitigate bias due to dataset shifts.^8^ Temporarily, data shortages and unfavourable model performance may be overcome by data augmentation techniques—in computer vision, image transformations, mirroring or rotation is common to increase training sample size—or generation of synthetic data, which closely mimic their underlying training data. Yet, overcoming the underlying challenge of data sharing and bias mitigation requires large‐scale collaborations. Investigators may also choose to train models using federated learning—where the model is shared between collaborators—or swarm learning—where only model weights are shared.

## EXPLAINABILITY IS ESSENTIAL TO VALIDATE OUTPUTS AND ENABLE DIGITAL BIOMARKER DISCOVERY

For both clinicians and patients to build trust in AI‐based CDSS, model outputs have not only to be accurate but also explainable. Deep learning models often outperform conventional machine learning models on images and language, yet are deemed ‘black boxes’ due to their opaque decision‐making. However, for structured data with lower complexity such as tabular data, simpler tree‐based or regression‐based models may deliver sufficient performance while offering better interpretability. An entire field of explainable AI has arisen aiming to decipher decision‐making processes (a comprehensive review for medical image analysis is provided by van der Velden et al.^9^). In medical computer vision, model attention can be traced to certain image areas highly relevant for classification.^10^, ^11^ This way, haematologists can ensure models evaluate medically relevant image areas instead of focusing on background or artefacts. Moreover, explainable AI may unveil digital biomarkers such as genotype–phenotype links as recently demonstrated for mutations of the spliceosome, *TET2* or monosomy 7 in myelodysplastic neoplasms^12^ or mutations of *NPM1* as well as *RUNX1::RUNX1T1*, *CBFB::MYH11* or *PML::RARA* fusions in acute myeloid leukaemia and acute promyelocytic leukaemia respectively.^13^, ^14^, ^15^ Thus, explainability may not only enable clinicians to further validate model outputs but also provide novel insights into disease biology via digital biomarkers.

## AI LITERACY IS A KEY REQUIREMENT TO FUTURE‐PROOF CLINICAL CARE

To effectively interpret AI outputs, haematologists should receive the necessary training. Otherwise, even well‐explained model outputs may lead to misinterpretation. Medical schools should integrate AI literacy into existing curricula by addressing AI applications and their limitations within relevant educational modules. For instance, during microscopy training, students could be introduced to AI‐supported tools for image classification, interpreting their outputs and recognising potential pitfalls. In residency programmes, the use of CDSS and the use of language models to help with documentation should be addressed. Importantly, this requires room within educational frameworks to accommodate additional content and instructors capable of delivering it. Yet, overreliance on diagnostic AI may lead to a decline in diagnostic skills—such as cytomorphology or flow cytometry interpretation—as an unintended consequence.^16^ Haematologists must balance automating time‐consuming tasks conveniently solved by AI on the one hand and identifying edge cases that yet cannot be accurately identified by AI on the other hand.

## THE ROAD AHEAD

AI‐based CDSS has come a long way from initially disappointing performances of systems such as IBM Watson. The development of the transformer^17^—a key component of large language models such as ChatGPT—has sparked a movement towards multimodal and generalist medical AI such as Articulate Medical Intelligence Explorer (AMIE) outperforming primary care physicians both in accurately identifying correct diagnoses as well as in conversational skills and empathy.^18^, ^19^ The prevailing belief that clinicians would only be supported by AI tools in the future is therefore increasingly challenged as models already outperform human experts across an expanding range of tasks. The current challenge lies in how to effectively integrate such systems into clinical practice and extend their capabilities to highly specialised domains such as haematology. Furthermore, the dynamic and evolving nature of AI algorithms challenges the assumptions of rigid diagnostic testing in regulatory frameworks such as the medical device regulation or in vitro diagnostic regulation (IVDR) of the European Union (EU). Especially generalist models such as commercially available chat bots with medical domain knowledge may pose a risk in the hands of medical experts untrained in AI or laypersons without proper regulatory guidance and oversight.^20^ Novel regulatory frameworks, such as the EU AI Act,^21^ must strike a balance between enforcing mandatory safeguards and quality controls to mitigate risk, while still fostering innovation and international collaboration.

## AUTHOR CONTRIBUTIONS

J‐NE and JMM conceptualised the work. J‐NE wrote the first draft and created the visual abstract. JMM revised and edited the manuscript.

## CONFLICT OF INTEREST STATEMENT

J‐NE declares consulting services for AstraZeneca, Novartis, Janssen. Furthermore, he holds shares in Cancilico, has received an institutional research grant from Novartis and has received honoraria from Amgen, AstraZeneca, Janssen, Novartis and Pfizer. JMM declares consulting services for Janssen, Roche, Gilead, Abbvie, Jazz, Pfizer, Astellas, Novartis, AstraZeneca and Clycostem. Furthermore, he holds shares in Cancilico and Synagen; has received institutional research grants from Janssen, Jazz and Novartis; and has received honoraria from Novartis, Roche, Janssen, Abbvie, Pfizer, Sanofi, Astellas and Beigene.
